# The Emergence of Antimicrobial Resistance and Virulence Characteristics in *Enterococcus* Species Isolated from Bovine Milk

**DOI:** 10.3390/antibiotics12081243

**Published:** 2023-07-28

**Authors:** Beatriz Rizzo Paschoalini, Karen Vanessa Munive Nuñez, Juliana Takahashi Maffei, Hélio Langoni, Felipe Freitas Guimarães, Clarice Gebara, Natylane Eufransino Freitas, Marcos Veiga dos Santos, Carlos Eduardo Fidelis, Roberto Kappes, Mônica Correia Gonçalves, Nathália Cristina Cirone Silva

**Affiliations:** 1 Department of Food Science and Nutrition, School of Food Engineering, University of Campinas, Campinas 13083-862, SP, Brazil; beatriz.paschoalini@unesp.br (B.R.P.); karen_vmn@hotmail.com (K.V.M.N.); julianatakahashimaffei@gmail.com (J.T.M.); 2 Department of Animal Production and Preventive Veterinary Medicine, Faculty of Veterinary Medicine and Animal Science, São Paulo State University, Botucatu 18618-681, SP, Brazil; helio.langoni@unesp.br (H.L.); felipefreitasguimaraes@hotmail.com (F.F.G.); 3 Food Research Center, School of Veterinary Medicine and Animal Science, Federal University of Goiás, Campus Road, Goiânia 74690-900, GO, Brazil; claricegebara@ufg.br (C.G.); natyllane@hotmail.com (N.E.F.); 4 Department of Animal Nutrition and Production, School of Veterinary Medicine and Animal Sciences, University of São Paulo (USP), Pirassununga 13635-900, SP, Brazil; mveiga@usp.br (M.V.d.S.); fidelis1999@hotmail.com (C.E.F.); 5 Center for Agroveterinary Sciences, University of the State of Santa Catarina, Lages 88520-000, SC, Brazil; roberto_kappes2.8@hotmail.com; 6 Center for Agro-Food Science and Technology, Federal University of Campina Grande, Campina Grande 58840-000, PB, Brazil; mnygoncalves@gmail.com

**Keywords:** *E. faecium*, *E. faecalis*, Vancomycin-resistant enterococci (VRE), virulence factors

## Abstract

*Enterococcus* spp., including *E. faecalis* and *E. faecium*, pose risks to dairy farms as opportunistic pathogens. The study evaluates antimicrobial resistance (AMR) and virulence characteristics of *Enterococcus* spp. isolated from bovine milk. Bile esculin agar was used to assess 1471 milk samples, followed by colony identification, gram staining, catalase tests, and 45 °C incubation. PCR analysis targeted *E. faecalis* and *E. faecium* in characteristic *Enterococcus* spp. colonies, with MALDI-TOF used for negative samples. Multiple tests, including disk diffusion, chromogenic VRE agar for vancomycin resistance, Vancomycin Etest^®^ for MIC determination, and PCR for virulence factors (*cyl*A, *esp*, *efa*A, *ace*, *asa*1, *gel*E, and *hyl* genes), were performed. Out of 100 identified strains, *E. durans* (30.66%), *E. faecium* (26.28%), and *E. faecalis* (18.25%) were predominant. AMR in *Enterococcus* spp. varied, with the highest rates against rifampicin (27%), tetracycline (20%), and erythromycin (18%). Linezolid (5%), vancomycin, ciprofloxacin, and teicoplanin (3% each) had lower prevalence. *E. faecium* and *E. faecalis* showed high AMR to rifampicin, erythromycin, and tetracycline. Thirty-two strains (18.98%) grew on VRE Chromoselect agar, while 4 (2 *E. faecalis* and 2 *E. faecium*) showed vancomycin resistance by MIC values. *E. faecalis* carried *gel*E (45.5%) and *asa*1 (36%), and *E. gallinarum* had 9.1% with the *asa*1 gene. Detecting resistant *Enterococcus* in bovine milk supports control strategies for enterococci on dairy farms, highlighting AMR concerns in the food chain.

## 1. Introduction

Bovine mastitis is considered an endemic disease, common and economically harmful to the dairy industry, causing physicochemical changes in milk that reflect its composition and quality [1,2,3]. Antibiotic treatment of mastitis is a common practice; however, it is common for pathogens to acquire antimicrobial resistance, AMR [4], reducing cure rates and selecting drug-resistant opportunistic pathogens [5], such as the *Enterococcus* genus [6].

The *Enterococcus* genus is recognized as an important opportunistic pathogen group, with *E. faecalis* and *E. faecium* being the most representative species, accounting for over 80% of isolates associated with infections. These two species have been identified as the third and fourth most prevalent nosocomial pathogens worldwide [7,8]. The genus is ubiquitous and can easily contaminate the food chain and affect intestinal colonization, including the human gut, where they usually are commensal microorganisms. When present in environments, they are indicators of faecal contamination in water [7,8,9]. Similarly to the human microbiota, the *Enterococcus* species most commonly found in the animal microbiota are *E. faecalis, E. faecium, E. hirae,* and *E. durans* [8]. However, many species from this genus can contaminate and colonize the teat skin, leading to bovine mastitis [4,10,11]. The growing number of fatalities attributed to these bacteria is a cause for concern. In 2019, many deaths were linked to AMR, with *E. faecalis* and *E. faecium* as causative agents. The estimated deaths associated with these bacteria ranged from 100,000 to 250,000 [12].

*Enterococcus* is commonly observed in the food industry, mainly in dairy products, attributed to its high tolerance to disadvantageous conditions, allowing it to survive in adverse environmental conditions [11]. The concerning emergence of multidrug-resistant enterococci and carriers of genes encoding virulence factors capable of evading the human immune system has been extensively researched [13,14,15]. *Enterococcus* spp. can be reservoirs of AMR and virulence genes [6,8], and the ease of transmission and perpetuation of virulence and resistance genes to other species and different bacterial genera make enterococcal infection a significant concern for public health [16]. Normal strains lacking genes for virulence factors may acquire virulence genes through horizontal gene transfer and spontaneous mutations [16,17]. The virulence factors, including aggregation substance (*asa*1), collagen-binding protein (*ace*), virulence factor associated with infective endocarditis (*efa*A), enterococcal surface protein associated with biofilm production (*esp*), gelatinase (*gel*E), cytolysin (*cyl*A), and hyaluronidase (*hyl*), play crucial roles in the invasion and spread of *Enterococcus*, thereby influencing its pathogenicity in bovine mastitis [11,17,18,19].

Vancomycin-resistant enterococci (VRE) are a global health concern due to their rapid spread and resistance to vancomycin, a critical antibiotic for severe Gram-positive bacterial infections. These multidrug-resistant bacteria pose risks to patient safety with increased morbidity and mortality [8,16,20]. While vancomycin acts by blocking cell wall formation in bacteria, enterococci have acquired genes that allow them to bypass the susceptible steps targeted by the antibiotic, leading to complex resistance mechanisms [8,20,21]. VRE has been detected in various samples, including milk, emphasizing the role of food in its dissemination [20,22]. Furthermore, inappropriate use of antimicrobials in animal feeding contributes to VRE prevalence. It leads to multidrug resistance and nosocomial infections, particularly in developing countries where controlling its spread is challenging due to limited resources [16,23].

The involvement of enterococci in bovine mastitis is the most common reason for using antibiotics in dairy cows. It is particularly interesting due to the misuse of antibiotics in cattle and the broad survival characteristics of *Enterococcus* species in the environment associated with the ease of transmission of virulence genes between strains [17,24]. Therefore, to assess the susceptibility profile of *Enterococcus* strains, a variety of antibiotics with different classes and mechanisms of antimicrobial action were selected. The chosen antibiotics included beta-lactams, glycopeptides, lipoglycopeptides, macrolides, tetracyclines, fluoroquinolones, nitrofurantoin, ansamycins, phenols, and oxazolidinones to cover a broad range of antimicrobial actions.

Due to the significant impact on public health, this research was conducted to examine the occurrence of *Enterococcus* spp. in bovine milk, with a particular focus on *E. faecalis* and *E. faecium*. The study also aimed to determine the prevalence of AMR profile (phenotypic), including vancomycin resistance and virulence genes (genotypic) among the identified *Enterococcus* strains. This knowledge is vital for developing effective control strategies in dairy farms, safeguarding public health, preserving the efficacy of antimicrobial agents, and ensuring the welfare and productivity of dairy cattle.

## 2. Results and Discussion

### 2.1. Prevalence of Enterococcus spp. in Bovine Milk

A total of 100 strains were identified as *Enterococcus*. The analysis revealed that *E. faecium* (26%), *E. faecalis* (22%), and *E. durans* (22%) were the most prevalent species among the identified isolates. These strains were sourced from different origins, including four samples obtained from cows with clinical mastitis, 34 with subclinical mastitis, 59 from healthy cows, and three samples that could not be classified into any of the above-mentioned categories.

Our study revealed a more significant frequency of *Enterococcus* in healthy samples than in those with clinical and subclinical mastitis. Several factors can explain this finding. *Enterococcus* is considered an opportunistic pathogen and a commensal microorganism commonly found in the normal gut flora of humans and animals. Therefore, its presence in milk samples is expected, even when mastitis is absent. Furthermore, the higher prevalence of *Enterococcus* in healthy samples can be attributed to its commensal nature, ability to survive in adverse conditions, and intrinsic resistance mechanisms [11,13]. Detailed information regarding the species distribution and sample sources is provided in Table 1.

### 2.2. Antimicrobial Resistance

Antimicrobial resistance testing was conducted to assess the resistance of the analyzed strains. A total of 13 strains (13%) were resistant to at least one antibiotic, 16 (16%) were identified as resistant to two antibiotics, and 13 (13%) were multi-resistant (resistant to three or more antibiotics). Additionally, three strains were found resistant to vancomycin in the disk test. Regarding growth on vancomycin-resistant enterococci (VRE) Chromoselect agar, 32 (18.98%) strains exhibited characteristic growth according to the manufacturer′s instructions.

Concerning the difference in antibiotic susceptibility among the same strains isolated from clinical mastitis, subclinical mastitis, and healthy samples, we observed a significant association between the type of mastitis and sensitivity to antibiotics, specifically vancomycin and teicoplanin. The proportion of sensitive isolates was higher in samples from healthy cows than those with subclinical mastitis for both antibiotics. Studies have shown that the composition and diversity of the milk microbiome can vary depending on the health status of the cows. Previous research has demonstrated that a balanced and diverse microbial community, including beneficial bacteria, characterizes the milk microbiome of healthy cows. In contrast, cows with mastitis may exhibit an altered milk microbiome with a higher abundance of potentially pathogenic bacteria that could contribute to reduced antibiotic sensitivity. It is essential to highlight that *Enterococcus* may be a part of the healthy milk microbiota [15,25,26].

Based on our results, the higher proportion of susceptible isolates for vancomycin and teicoplanin in healthy samples compared to clinical and subclinical samples may be attributed to a more favorable microbial composition in the milk from healthy cows that potentially enhance the effectiveness of the treatment. However, it is essential to note that these results may not necessarily extend to other antibiotics, as no significant association was observed between the type of mastitis and sensitivity for the other antibiotics tested. In addition, individual variations, such as farm management practices, immune response, and genetic variations, could contribute to the observed differences in antibiotic susceptibility among the different milk sources [15,22].

**Figure 1:**
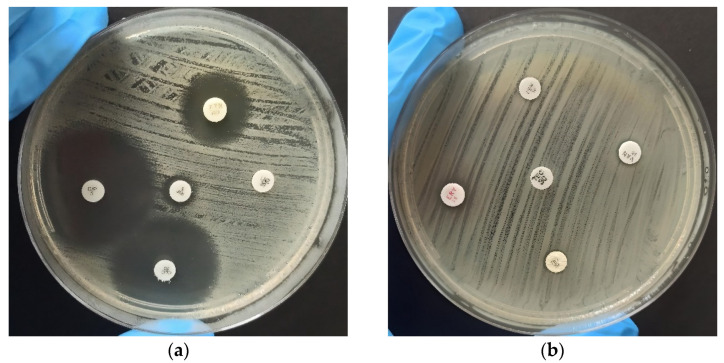
Antibiogram for evaluation of antimicrobial susceptibility of identified *Enterococcus* species using the disk diffusion method. The strains were categorized as susceptible (S), intermediate (I), or resistant (R). (**a**) Displays the results of the test conducted using Ciprofloxacin (S), Nitrofurantoin (I), Rifampicin (R), Chloramphenicol (S), and Linezolid (R) antibiotics. (**b**) Displays the assessment of Penicillin (R), Vancomycin (R), Teicoplanin (R), Erythromycin (R), and Tetracycline (R) antibiotics.

The analysis of antimicrobial resistance was performed using the disk diffusion method, as shown in Figure 1, which clearly illustrates the distinction between antimicrobial resistance (Figure 1a) and susceptibility (Figure 1b) among the analyzed *Enterococcus* strains. Zone diameters were then interpreted and categorized according to the Clinical and Laboratory Standards Institute (CLSI M100-S24) breakpoints [27], classifying them as susceptible, intermediate, or resistant. The detailed results of this analysis are displayed in Table 2. Among the 100 isolates, the highest resistance rates were observed against rifampicin (27%), followed by tetracycline (20%), erythromycin (18%), penicillin, and nitrofurantoin (11%). Resistance rates were lower for linezolid (5%), vancomycin, ciprofloxacin, and teicoplanin (3% each).

The antimicrobial resistance testing results revealed distinct patterns among different *Enterococcus* species. The *E. faecalis* strains exhibited resistance to tetracycline (27.3%), erythromycin (22.7%), and rifampicin (13.6%), while all strains displayed susceptibility to chloramphenicol and nitrofurantoin. On the other hand, a higher prevalence of resistance was observed in *E. faecium* strains, with 42.3% being resistant to rifampicin, 50% to erythromycin, and 46.2% to tetracycline. Notably, resistance to ciprofloxacin and chloramphenicol was not detected. Our results were similar to those reporting a higher prevalence of antibiotic resistance in *E. faecium* than in *E. faecalis* [28,29], highlighting the importance of surveillance and control of antibiotic resistance in *Enterococcus* species.

In addition, among the other five species of enterococci identified, *E. durans* exhibited the highest resistance rates to the tested antimicrobials, with 59.1% of isolates being resistant to rifampicin, 40.9% to nitrofurantoin, 4.5% to ciprofloxacin, and 4.5% to linezolid. No antibiotic resistance was identified in *E. gallinarum* and *E. saccharolyticus,* except for two strains resistant to tetracycline and one to ciprofloxacin, respectively. All *E. hirae and E. casseliflavus* isolates showed sensitivity to the ten antibiotics evaluated.

In our study, tetracycline demonstrated notable efficacy against *E. faecalis* and *E. faecium*, emphasizing its importance as an effective antimicrobial agent. These findings align with a previous study by Yang et al. [6], which evaluated the antimicrobial resistance of *E. faecalis* isolated from bovine mastitis cases in China and found high rates of resistance to tetracycline (87.7%) and erythromycin (79%). These results underscore the importance of tetracycline as an essential tool in combating *Enterococcus* infections.

Erythromycin resistance was found in 22.7% of *E. faecalis* strains and 50% in *E. faecium*. The study by Nasaj et al. [30] supports our findings, as it reported 62.3% of *E. faecalis* and 86.6% of *E. faecium* as resistant to the antibiotic. On the other hand, Rózańska et al. [11] observed a higher proportion of erythromycin-resistant *E. faecalis* strains (50.57%) compared to *E. faecium* (31.43%) in Polish dairy farms. Additionally, previous research has suggested a potential association between the high levels of erythromycin resistance and the widespread use of the antibiotic in animal husbandry [31].

In our study, the frequency of rifampicin-resistant strains was 13.6% in *E. faecalis* and 42.3% in *E. faecium*, the highest value among the tested antimicrobials. While rifampicin is not routinely used to treat infections caused by *E. faecium*, the species commonly present acquired resistance to this antimicrobial [32]. Deshpande et al. [33] provided supporting evidence for our findings by reporting resistance rates to rifampicin of 5.9% in *E. faecalis* and 65.9% in *E. faecium* among strains isolated in the USA. In Europe, the rates were even higher, with 21.4% of *E. faecalis* and 67.5% of *E. faecium*. Yang et al. [6] identified 18.5% of *E. faecalis* strains resistant to rifampicin; Jahansepas et al. [29] isolated enterococci from human body fluids, predominantly *E. faecalis* and *E. faecium*, with resistance rates of 71.2% and 94.3%, respectively. Furthermore, Sharifi et al. [34] reported complete resistance to rifampicin in *E. faecium* (100%) and a substantial proportion in *E. faecalis* (81.2%), which supports our finding of higher resistance to rifampicin in *E. faecium* strains.

We observed that 34.6% of *E. faecium* and 9.1% of *E. faecalis* were resistant to penicillin. Depending on the species, Enterococci exhibit intrinsic resistance or reduced natural susceptibility to penicillins by expressing low-affinity penicillin-binding proteins (PBPs) that bind weakly to beta-lactam antibiotics [8,35]. Enterococci without intrinsic resistance to penicillins can develop acquired resistance, which is more common in *E. faecium* and rarer in *E. faecalis* [36,37]. Our study corroborates the results obtained by Rózańska et al. [11], where *E. faecium* exhibited higher penicillin resistance (5.71%) compared to *E. faecalis* (3.43%).

Chloramphenicol showed efficacy against all strains (100%), and ciprofloxacin was efficient against 97 strains (97%), in contrast to previous studies [6], where 14.8% of *E. faecalis* displayed resistance to chloramphenicol and 11.1% to ciprofloxacin. However, Rózańska et al. [11] reported a higher frequency of chloramphenicol-resistant strains, with 49.43% in *E. faecalis* and 20% in *E. faecium.* Interestingly, their findings on the efficacy of ciprofloxacin appear to be consistent with our results, showing only 0.57% of resistance in *E. faecalis* and 2.86% in *E. faecium.* However, it is essential to note that ciprofloxacin exhibits only moderate activity against enterococci and is not the first treatment choice [38]. Nitrofurantoin was also effective against 96% *E. faecalis,* and 8.33% *E. faecium* exhibited resistance to the antimicrobial. Notably, these results are consistent with those reported by Nasaj et al. [30], which showed 100% efficacy against *E. faecalis* and 25.4% resistance in *E. faecium.*

Resistant strains of *E. durans* to some of the evaluated antibiotics were found, as shown in Table 2. Our findings of *E. durans* resistance to rifampicin (*n* = 8) and nitrofurantoin (*n* = 13) align with the data from Výrostková et al. [24], which evaluated goat and sheep cheeses and reported that 100% and 66.7% of E. durans isolates showed resistance to rifampicin and nitrofurantoin, respectively. These results highlight the significance of *E. durans* as a species of interest and concern for public, animal, and dairy health.

In our study, linezolid demonstrated high efficacy against *E. faecalis* (95.5%) and *E. faecium* (88.5%) strains. Likewise, Nasaj et al. [30] obtained similar results, reporting linezolid′s effectiveness against 100% of both species, while Rózańska et al. [11] revealed resistance to this antimicrobial in 4% of *E. faecalis* strains and 2.86% of *E. faecium* strains. Despite the initial approval of Linezolid by the Food and Drug Administration (FDA) in 2000, the emergence of VREs quickly followed by identifying these strains in the United States in 2001 and subsequently in the United Kingdom in 2002. Their emergence is linked to genetic mutations and methylation events that affect the gene involved in the antimicrobial response [15].

Regarding teicoplanin, 4.5% *E. faecalis* and 7.7% *E. faecium* showed resistance. The study by Nasaj et al. [30] agrees with our results revealing higher resistance of *E. faecium* (73%) to the antibiotic regarding *E. faecalis* (5%), although with a more significant value disparity. Our prevalence results are similar to the study by Fernandes and Dhanashree [28], which reported 1.2% *E. faecalis* and 5.8% *E. faecium* resistant to the antibiotic. Notably, teicoplanin resistance shares the gene responsible for vancomycin resistance (*van*A), raising concerns regarding public health implications due to the frequency of gene mutations and the potential for horizontal transfer among enterococci [39].

Because of the results of VRE, usually accompanied by resistance to other antibiotics, the World Health Organization (WHO) has defined vancomycin-resistant *E. faecium* as multidrug-resistant organisms with high priority for surveillance [40], given that they have been extensively described in hospitals in recent decades [8]. Among our findings, it was possible to identify VRE through antibiogram results and by observing the growth of colonies on VRE Chromoselect Agar in 32 strains. We found three multi-resistant strains on Mueller-Hinton agar, constituting 3%. These strains included two *E. faecium* and one *E. faecali*s strain. Corroborating the WHO definitions and findings by Cortés et al. [41] and Mannu et al. [42], 100% of VRE strains identified from Mueller-Hinton agar were resistant to at least five other antibiotics.

Previous studies in the European Union and the United States have identified a higher prevalence of vancomycin-resistant *E. faecium* compared to *E. faecalis*. These studies also highlight an increase in the majority of both species over time [8,39,41]. Notably, the increase in the VRE prevalence in different countries has primarily been attributed to the incidence and spread of *van*A and *van*B genes accompanied by other virulence factors [21,30], which may be easily shared between species of the genus and, thus, may explain the increased incidence of vancomycin-resistant *E. faecalis* in recent years [8,39], as well as the VRE findings in the other species found in our study [43].

**Figure 2:**
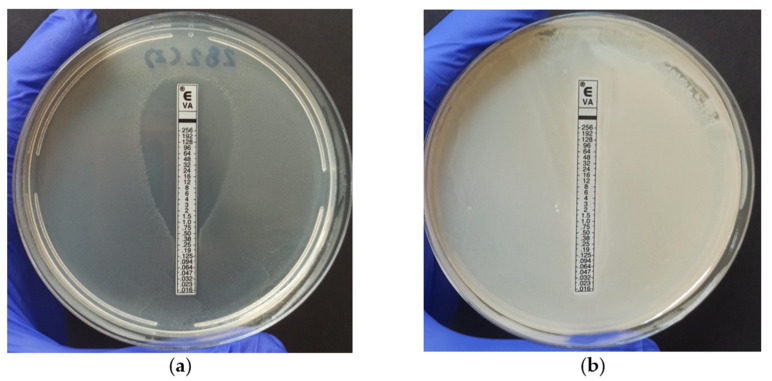
Vancomycin MIC determination using E-test^®^: (**a**) Demonstrates MIC values (0.38 µg/mL) below the CLSI-defined resistance threshold (≥32 µg/mL); (**b**) Characteristically exhibits vancomycin antimicrobial resistance (>256 µg/mL).

The Vancomycin Etest^®^ was performed to evaluate the Minimum Inhibitory Concentration (MIC) required to inhibit the growth of *E. faecium* and *E. faecalis,* which were identified as antimicrobial-resistant strains in the previously discussed tests. Figure 2 provides a visual representation of the results of the test. Out of the 36 strains tested, 26 (72.2%) exhibited sensitivity to the antibiotic (Figure 2a), while four strains, including two *E. faecalis* and two E. *faecium*, were classified as resistant to vancomycin as evident by halo formation at a concentration of ≥32 µg/mL (Figure 2b) [27]. Notably, 100% of the strains that showed resistance to vancomycin were resistant to 6 or 7 antibiotics in the antimicrobial susceptibility test, corroborating the WHO definition when considering VRE as a multi-resistance factor [40]. However, it is worth noting that the use of this method for determining the MIC has been relatively limited in the existing literature.

The Enterococcus genus has become a significant public health concern due to its role in causing nosocomial infections, including Healthcare-associated infections (HAIs)*,* primarily caused by *E. faecium* and *E. faecalis* [44]. The high incidence of HAIs caused by VRE and the increased number of antibiotic-resistant isolates found in recent years are of particular concern. For instance, in Germany, the percentage of vancomycin-resistant *E. faecium* strains increased from 10.5% in 2015 to 23.8% in 2018 [45], with some hospitals reporting as high as 80% of *E. faecium* isolates being resistant to vancomycin [15] constituting a significant concern for healthcare systems.

### 2.3. Detection of Virulence Genes

Virulence factors play a vital role in the pathogenesis of enterococci [18]. Previous research has highlighted the presence of various virulence genes in *E. faecalis* [46,47,48]. However, it is noteworthy that these genes are more commonly found in *E. faecium*. Table 3 provides a comprehensive overview of the prevalence of these genes among the tested *Enterococcus* species.

Various virulence factors have been attributed to playing different roles in the pathogenic potentials of *Enterococcus* spp. The *gel*E (45.5%) and *asa*1 (45.1%) genes were the only detected virulence genes in *E. faecalis*. For *E. gallinarum*, only one strain (9.1%) harbored the *asa*1 gene. None of the virulence genes were detected among *E. faecium* strains. As shown in this study, the most prevalent virulence gene for *E. faecalis* was the *gel*E gene which is involved in gelatinase production and enhances the survival of *Enterococcus* spp. The *asa*1 (aggregation substance-encoding gene) plays a significant role in enterococcal virulence. It enables the transfer of plasmids containing the sex pheromone gene and enhances virulence by promoting adherence to renal, cardiac, and intestinal epithelial cells [18]. A study on mastitis and normal raw bovine milk revealed that *E. faecalis* harboring *gel*E (85.7%) and *asa*1 (71.4%) might be causative agents for mastitis disease. The study highlighted that bovine mastitis milk exhibited higher virulence properties than normal raw milk, emphasizing the clinical significance of these virulence factors [17]. Likewise, our findings are consistent with those of Jahansepas et al. [29], in which a higher prevalence of *gel*E and *asa*1 was identified (88% and 74.4% of *E. faecalis*, respectively) compared to our results. Another study by Song et al. [47] found *gel*E and *asa*1 genes in 88% and 44% of *E. faecalis* strains, respectively.

On the other hand, Kiruthiga et al. [18] revealed that *asa*1 was the most common in *E. faecalis* among the five analyzed, followed by *gel*E, while *gel*E was more prevalent in *E. faecium*, followed by *esp*. In general, *gel*E and *asa*1 were significantly more common in *E. faecalis* when compared to *E. faecium*. The prevalence of virulence genes could enhance enterococcal infections and contribute to their pathogenesis. The evaluated strains in our study did not show the presence of *cyl*A, *esp*, *efa*A, *ace*, and *hyl* genes. In contrast, Jahansepas et al. [29] found a higher frequency of the *esp* and *hyl* genes in *E. faecium* than in *E. faecalis* strains. These differences in gene prevalence highlight the genetic diversity and variability within the *Enterococcus* species, which may have implications for their virulence and clinical outcomes.

Taken together, the findings of this study highlight the importance of understanding the prevalence and potential risks associated with *Enterococcus* in dairy environments. Furthermore, identifying antibiotic resistance and virulence factors in dairy *Enterococcus* raises concerns about the possible transfer of these traits to human pathogens, posing a significant threat to public health. Therefore, it is crucial to establish stringent control and monitoring measures to ensure milk quality and mitigate the spread of antibiotic resistance and virulence genes. In addition, future research should investigate the mechanisms of resistance and virulence gene transfer, specifically in dairy *Enterococcus*, aiming to develop strategies that minimize their dissemination. By addressing these issues, effective interventions can be developed to safeguard public health and enhance the safety of dairy products.

## 3. Materials and Methods

### 3.1. Identification of Enterococcal Isolates

A total of 1471 milk samples were collected from various herds in five distinct regions of Brazil, including São Paulo, Pará, Santa Catarina, Goiás, and Paraíba. The collection period was from March 2021 to February 2022. Among these samples, 720 were from healthy cows, 198 were from cows with clinical mastitis, 529 were from cows with subclinical mastitis, two were from farm bulk tanks, and 22 samples could not be classified accurately. The isolation was performed using the spread plate method with 100 μL and 10 µL, which were seeded with an L loop on the surface of Bile esculin azide agar (HiMedia) and incubated at 37 °C for 24 h. *Enterococcus* spp. were identified according to the results of the catalase-negative test (3% hydrogen peroxide), Gram stain (Gram-positive), and microscopic morphologic tests (cocci or short rod bacteria). Also, isolates were incubated in BHI broth at 45 °C for 48 h to select based on a temperature tolerance test. Strains exhibiting similar characteristics to *Enterococcus* were subjected to conventional PCR targeting *E. faecalis* and *E. faecium.* In cases where positive bands for both species were not detected, the samples were further analyzed using MALDI-TOF for accurate identification.

### 3.2. Identification of E. faecium and E. faecalis by PCR

DNA extraction was performed using the “InstaGene Matrix” kit (BIO-RAD) following the manufacturer′s instructions. For each strain, 1 mL aliquot of BHI broth was added in a sterile 1.5 mL microtube and centrifuged at 12,000 rpm for 1 min at room temperature. The supernatant was carefully discarded, and 200 µL of the matrix solution was added to the pellet. Microtubes were placed in a water bath at 60 °C for 25 min, homogenized on a shaker, inserted into a boiling water bath at 100 °C for 8 min, and homogenized once again, concluding the extraction. Finally, the resulting material was quantified using a NanoDrop Lite (ThermoFisher, Waltham, MA, USA).

PCR reactions were performed in a thermocycler SimpliAmp (ThermoFisher, Marsiling, Singapore), and GoTaq^®^ kit reagents (Promega, Madison, WI, USA) were used in the amounts described by Jackson, Fedorka-Cray, and Barrett [49] with some modifications. For detection of *E. faecium*, the final reaction volume was 25 µL containing 10 µL of buffer, 4 µL of MgCl_2_, 0.5 µL of dNTPs, 1.5 µL of Primer Forward FM1 (5′–GAAAAAACAATAGAAGAATTAT–3′), 1.5 µL of Primer Reverse FM2 (5′–TGCTTTTTTGAATTCTTCTTTA–3′), 0.4 µL of Taq DNA Polymerase, 2.1 µL of ultrapure water and 5 µL of sample DNA. For detection of *E. faecalis*, the final reaction volume was 25 µL containing 10 µL of buffer, 2.5 µL of MgCl_2_, 0.5 µL of dNTPs, 1.5 µL of Primer Forward FL1 (5′–ACTTATGTGACTAACTTAACC–3′), 1.5 µL of Primer Reverse FL2 (5′–TAATGGTGAATCTTGGTTTGG–3′), 0.3 µL of Taq DNA Polymerase, 3.7 µL of ultrapure water and 5 µL of sample DNA. For both species, the cycle profile used was initial denaturation at 95 °C for 4 min, 30 cycles of denaturation at 95 °C for 30 s, annealing at 55 °C for 1 min, and extension at 72 °C for 1 min, followed by a final extension at 72 °C for 7 min. Subsequently, PCR products were analyzed by electrophoresis on a 2% Tris-acetate-EDTA agarose gel containing SYBR^®^ Safe (Invitrogen, Carlsbad, CA, USA) and a 100 bp molecular weight DNA ladder (New England Biolabs, Ipswich, MA, USA) was used to validate the length of the products.

### 3.3. Identification of Enterococcus spp. by MALDI-TOF MS

Strains that grew in BHI at 45 °C for 48 h, along with bubble formation in the catalase test and/or that grew in VRE chromogenic medium but could not be confirmed as either *E. faecium,* or *E. faecalis* through PCR were sent to the Milk Quality Research Laboratory of the University of São Paulo (FMVZ–USP) for identification using Matrix-Assisted Laser Desorption Ionization Time Of Flight Mass Spectrometry (MALDI-TOF MS) for strains suspected to belong to the genus *Enterococcus*, according to Barcelos et al. [50].

### 3.4. Antimicrobial Resistance

The strains confirmed for the genus through PCR or MALDI-TOF analysis were inoculated onto plates containing VRE Chromoselect Agar Base (Sigma-Aldrich, São Paulo, SP, Brazil) to assess the emergence of VRE [51]. In addition, the antimicrobial susceptibility profiles of the strains were determined using the disk diffusion method.

For inoculum preparation, isolates were reactivated from cryopreservation in BHI broth at 37 °C for 24 h and standardized in saline solution to achieve a density equivalent to 0.5 on the McFarland scale and seeded in Mueller-Hinton medium using swabs. Subsequently, Petri dishes (diameter: 90 mm) were used, and antibiotics discs were placed with sterile forceps [52]. The antimicrobial agents evaluated, and their corresponding concentrations were as follows: penicillin (10 U), vancomycin (30 mg), teicoplanin (30 mg), erythromycin (15 µg), tetracycline (30 µg), ciprofloxacin (5 µg), nitrofurantoin (300 µg), rifampicin (5 µg), chloramphenicol (30 µg) and linezolid (30 µg). Finally, the inoculated plates were incubated at 37° for 24 h, and inhibition halos were measured after this period. The diameters of the zone of inhibition were interpreted according to the CLSI M100-S24 [27].

The *E. faecium* and *E. faecalis* strains confirmed to be resistant to vancomycin in the disk diffusion test were submitted to Etest^®^ (BioMérieux, Rio de Janeiro, Brazil). The strains were seeded on the surface of plates containing Mueller-Hinton agar, and a strip impregnated with increasing antibiotic concentrations was placed above it. After incubation at 37° for 24 and 48 h, the Minimum Inhibitory Concentration values were determined following the guidelines outlined by CLSI M100-S24 [27]. In addition, the study also included the evaluation of VRE strains targeting *E. gallinarum* and *E. hirae*.

### 3.5. Virulence Genes

The analysis of virulence genes was carried out through Conventional PCR. The selection of *cyl*A, *esp*, *efa*A, *ace*, *asa*1, *gel*E, and *hyl* genes was based on their known association with virulence and relevance as indicators of pathogenic potential in *Enterococcus* species. A final reaction volume of 25 µL was prepared, containing 10 µL of buffer, 2.5 µL of MgCl_2_, 0.5 µL of dNTPs, 1.5 µL of Primer Forward, 1.5 µL of Primer Reverse, 0.25 µL of Taq DNA Polymerase, 3.75 µL of ultrapure water, and 5 µL of sample DNA. The specific primer sequences used for each gene can be found in Table 4.

## 4. Conclusions

We identified several species of *Enterococcus*, including *E. faecium*, *E. faecalis*, *E. hirae, E. gallinarum*, *E. saccharolyticus,* and *E. casseliflavus*. *E. faecium* was the most prevalent among these species within the *Enterococcus* genus. Virulence determinants were more common in *E. faecalis,* which harbored *gel*E and *asa*1 genes; however, virulence genes in *E. faecium* were absent. Considering the vancomycin resistance, we found the presence in our strains, emphasizing the importance of the control of AMR in food processing chains. In addition, the antibiotic resistance observed against rifampicin, tetracycline, erythromycin, and nitrofurantoin highlights the potential concern of these strains as reservoirs for antibiotic resistance genes.

## Figures and Tables

**Table 1:** Frequency of *Enterococcus* species isolated from bovine milk.

Species	Isolation (*n* = 100)	Frequency (%)	Sample Type			
			Clinical Mastitis (*n* = 4)	Subclinical Mastitis (*n* = 34)	No Mastitis (Healthy) (*n* = 59)	Not Classified (*n* = 3)
*E. casseliflavus*	1	1	0	0	1	0
*E. durans*	22	22	1	2	18	1
*E. faecalis*	22	22	1	11	9	1
*E. faecium*	26	26	2	8	15	1
*E. gallinarum*	11	11	0	5	6	0
*E. hirae*	13	13	0	4	9	0
*E. saccharolyticus*	5	5	0	4	1	0
Total	100	100	4	34	59	3

**Table 2:** Antimicrobial resistance of *Enterococcus* species isolated from bovine milk.

Distribution of Strains that Displayed Susceptibility, Intermediate Resistance, or Resistance (%)																					
Antimicrobial ^1^	*E. faecalis* (*n* = 22)			*E. faecium* (*n* = 26)			*E. durans* (*n* = 22)			*E. hirae* (*n* = 13)			*E. casseliflavus* (*n* = 1)			*E. gallinarum* (*n* = 11)			*E. saccharolyticus* (*n* = 5)		
	S	I	R	S	I	R	S	I	R	S	I	R	S	I	R	S	I	R	S	I	R
PEN	90.9	0	9.1	65.4	0	34.6	100	0	0	100	0	0	100	0	0	100	0	0	100	0	0
VAN	95.5	0	4.5	92.3	0	7.7	100	0	0	100	0	0	100	0	0	100	0	0	100	0	0
TEI	95.5	0	4.5	92.3	0	7.7	100	0	0	100	0	0	100	0	0	100	0	0	100	0	0
ERY	63.6	13.6	22.7	46.2	3.8	50	100	0	0	69.2	30.8	0	100	0	0	100	0	0	80	20	0
TET	68.2	4.5	27.3	50	3.8	46.2	90.9	9.1	0	92.3	7.7	0	100	0	0	9.1	72.7	18.2	80	20	0
CIP	77.3	18.2	4.5	84.6	15.4	0	95.5	0	4.5	100	0	0	100	0	0	36.4	63.6	0	80	0	20
NIT	100	0	0	92.3	0	7.7	59.1	0	40.9	100	0	0	100	0	0	100	0	0	100	0	0
RIF	72.7	13.6	13.6	30.8	26.9	42.3	36.4	4.5	59.1	100	0	0	100	0	0	90.9	9.1	0	100	0	0
CHL	100	0	0	96.2	3.8	0	95.5	4.5	0	100	0	0	100	0	0	90.9	9.1	0	100	0	0
LIN	95.5	0	4.5	88.5	0	11.5	81.8	13.6	4.5	100	0	0	100	0	0	100	0	0	100	0	0

^1^ PEN = penicillin, VAN = vancomycin, TEI = teicoplanin, ERY = erythromycin, TET = tetracycline; CIP = ciprofloxacin; NIT = nitrofurantoin, RIF = rifampicin CHL = chloramphenicol; LIN = linezolid; S = susceptible; R = resistant; I = intermediate; %: were calculated according to the *n* of tested species.

**Table 3:** Distribution of virulence genes among *Enterococcus* species from bovine milk.

Species	Virulence Genes (%)						
	*cyl*A	*esp*	*efa*A	*ace*	*asa*1	*gel*E	*hyl*
*E. caseliflavus* (*n* = 1)	-	-	-	-	-	-	-
*E. durans* (*n* = 22)	-	-	-	-	-	-	-
*E. faecalis* (*n* = 22)	-	-	-	-	8 (36%)	10 (45.5%)	-
*E. faecium* (*n* = 26)	-	-	-	-	-	-	-
*E. gallinarum* (*n* = 11)	-	-	-	-	1 (9.1%)	-	-
*E. hirae* (*n* = 13)	-	-	-	-	-	-	-
*E. saccharolyticus* (*n* = 5)	-	-	-	-	-	-	-

-, =absence of virulence genes.

**Table 4:** Virulence gene primers for *E. faecium* and *E. faecalis* were used in the study.

Gene	Primers Sequence (5′ to 3′) ^1^	Product Size (bp)	Reference
*gel*E	*gelE-F* TATGACAATGCTTTTTGGGAT	213	[53]
	*gelE-R* AGATGCACCCGAAATAATATA		
*hyl*	*hyl-F* ACAGAAGAGCTGCAGGAAATG	276	[53]
	*hylR* GACTGACGTCCAAGTTTCCAA		
*asa*1	*asa1-F* GCACGCTATTACGAACTATGA	375	[53]
	*asa1-R* AAGAAAGAACATCACCACGA		
*esp*	*esp-F* AGATTTCATCTTTGATTCTTGG	510	[53]
	*esp-R* AATTGATTCTTTAGCATCTGG		
*cyl*A	*cylA-F* ACTCGGGGATTGATAGGC	688	[53]
	*cylA-R* GCTGCTAAAGCTGCGCTT		
*efa*A	*efaA-F* GACAGACCCTCACGAATA	705	[54]
	*efaA-R* AGTTCATCATGCTGTAGTA		
*ace*	*ace-F* GGAATGACCGAGAACGATGGC	616	[55]
	*ace-R* GCTTGATGTTGGCCTGCTTCCG		

^1^ F: forward; R: reverse

## Data Availability

All data generated or analyzed during this study are included in this published article in the main manuscript.

## References

[1] Reshi A.A., Husain I., Bhat S.A., Rehman M.U., Razak R., Bilal S., Mir M.R. (2015). Bovine Mastitis as an Evolving Disease and Its Impact on the Dairy Industry. Int. J. Curr. Res. Rev..

[2] Ruegg P.L. (2017). A 100-Year Review: Mastitis detection, management, and prevention. J. Dairy Sci..

[3] Gonçalves J.L., Kamphuis C., Martins C.M.M.R., Barreiro J.R., Tomazi T., Gameiro A.H., Hogeveen H., dos Santos M.V. (2018). Bovine subclinical mastitis reduces milk yield and economic return. Livest. Sci..

[4] Dyson R., Charman N., Hodge A., Rowe S., Taylor L. (2022). A survey of mastitis pathogens including antimicrobial susceptibility in southeastern Australian dairy herds. J. Dairy Sci..

[5] Sharun K., Dhama K., Tiwari R., Gugjoo M.B., Yatoo M.I., Patel S.K., Pathak M., Karthik K., Khurana S.K., Singh R. (2021). Advances in therapeutic and managemental approaches of bovine mastitis: A comprehensive review. Veter.-Q..

[6] Yang F., Zhang S., Shang X., Wang X., Yan Z., Li H., Li J. (2019). Short communication: Antimicrobial resistance and virulence genes of *Enterococcus faecalis* isolated from subclinical bovine mastitis cases in China. J. Dairy Sci..

[7] Zhong Z., Zhang W., Song Y., Liu W., Xu H., Xi X., Menghe B., Zhang H., Sun Z. (2017). Comparative genomic analysis of the genus *Enterococcus*. Microbiol. Res..

[8] Torres C., Alonso C.A., Ruiz-Ripa L., León-Sampedro R., Del Campo R., Coque T.M. (2018). Antimicrobial Resistance in *Enterococcus* spp. of animal origin. Microbiol. Spectr..

[9] Eggesbø M., Moen B., Peddada S., Baird D., Rugtveit J., Midtvedt T., Bushel P.R., Sekelja M., Rudi K. (2011). Development of gut microbiota in infants not exposed to medical interventions. Apmis.

[10] de Oliveira R.P., Aragão B.B., de Melo R.P.B., da Silva D.M.S., de Carvalho R.G., Juliano M.A., Farias M.P.O., de Lira N.S.C., Mota R.A. (2022). Bovine mastitis in northeastern Brazil: Occurrence of emergent bacteria and their phenotypic and genotypic profile of antimicrobial resistance. Comp. Immunol. Microbiol. Infect. Dis..

[11] Różańska H., Lewtak-Piłat A., Kubajka M., Weiner M. (2019). Occurrence of enterococci in mastitic cow’s milk and their antimicrobial resistance. J. Veter.-Res..

[12] Murray C.J.L., Ikuta K.S., Sharara F., Swetschinski L., Aguilar G.R., Gray A., Han C., Bisignano C., Rao P., Wool E. (2022). Global burden of bacterial antimicrobial resistance in 2019: A systematic analysis. Lancet.

[13] Lebreton F., Willems R.J.L., Gilmore M.S. (2014). Enterococcus Diversity, Origins in Nature, and Gut Colonization.

[14] Frieri M., Kumar K., Boutin A. (2017). Antibiotic resistance. J. Infect. Public Health.

[15] Selleck E.M., Van Tyne D., Gilmore M.S. (2019). Pathogenicity of enterococci. Microbiol. Spectr..

[16] Raza T., Ullah S.R., Mehmood K., Andleeb S. (2018). Vancomycin resistant Enterococci: A brief review. J. Pak. Med. Assoc..

[17] Kim H.-J., Youn H.-Y., Kang H.-J., Moon J.-S., Jang Y.-S., Song K.-Y., Seo K.-H. (2022). Prevalence and Virulence Characteristics of *Enterococcus faecalis* and *Enterococcus faecium* in Bovine Mastitis Milk Compared to Bovine Normal Raw Milk in South Korea. Animals.

[18] Kiruthiga A., Padmavathy K., Shabana P., Naveenkumar V., Gnanadesikan S., Malaiyan J. (2020). Improved detection of esp, hyl, asa1, gelE, cylA virulence genes among clinical isolates of Enterococci. BMC Res. Notes.

[19] Aung M.S., Urushibara N., Kawaguchiya M., Ohashi N., Hirose M., Kudo K., Tsukamoto N., Ito M., Kobayashi N. (2023). Antimicrobial Resistance, Virulence Factors, and Genotypes of *Enterococcus faecalis* and *Enterococcus faecium* Clinical Isolates in Northern Japan: Identification of *optrA* in ST480 *E. faecalis*. Antibiotics.

[20] El-Zamkan M.A., Mohamed H.M.A. (2021). Antimicrobial resistance, virulence genes and biofilm formation in *Enterococcus* species isolated from milk of sheep and goat with subclinical mastitis. PLoS ONE.

[21] Stogios P.J., Savchenko A. (2020). Molecular mechanisms of vancomycin resistance. Protein Sci..

[22] Yoon S., Lee Y.J. (2021). Molecular Characteristics of *Enterococcus faecalis* and *Enterococcus faecium* from Bulk Tank Milk in Korea. Animals.

[23] Margalho L.P., van Schalkwijk S., Bachmann H., Sant’ana A.S. (2020). *Enterococcus* spp. in Brazilian artisanal cheeses: Occurrence and assessment of phenotypic and safety properties of a large set of strains through the use of high throughput tools combined with multivariate statistics. Food Control.

[24] Výrostková J., Regecová I., Dudriková E., Marcinčák S., Vargová M., Kováčová M., Maľová J. (2021). Antimicrobial Resistance of *Enterococcus* sp. Isolated from Sheep and Goat Cheeses. Foods.

[25] Addis M.F., Tanca A., Uzzau S., Oikonomou G., Bicalho R.C., Moroni P. (2016). The bovine milk microbiota: Insights and perspectives from -omics studies. Mol. Biosyst..

[26] Kaczorowski Ł., Powierska-Czarny J., Wolko Ł., Piotrowska-Cyplik A., Cyplik P., Czarny J. (2022). The Influence of Bacteria Causing Subclinical Mastitis on the Structure of the Cow’s Milk Microbiome. Molecules.

[27] (2022). Performance Standards for Antimicrobial Testing Susceptibility.

[28] Fernandes S.C., Dhanashree B. (2013). Drug resistance & virulence determinants in clinical isolates of *Enterococcus* species. Indian J. Med. Res..

[29] Jahansepas A., Aghazadeh M., Rezaee M.A., Hasani A., Sharifi Y., Aghazadeh T., Mardaneh J. (2018). Occurrence of *Enterococcus faecalis* and *Enterococcus faecium* in Various Clinical Infections: Detection of Their Drug Resistance and Virulence Determinants. Microb. Drug Resist..

[30] Nasaj M., Mousavi S.M., Hosseini S.M., Arabestani M.R. (2016). Prevalence of Virulence Factors and Vancomycin-resistant Genes among *Enterococcus faecalis* and *E. faecium* Isolated from Clinical Specimens. Iran. J. Public Health.

[31] Aslam M., Diarra M.S., Service C., Rempel H. (2010). Characterization of Antimicrobial Resistance in *Enterococcus* Spp. Recovered from a Commercial Beef Processing Plant. Foodborne Pathog. Dis..

[32] Enne V.I., Delsol A.A., Roe J.M., Bennett P.M. (2004). Rifampicin resistance and its fitness cost in *Enterococcus faecium*. J. Antimicrob. Chemother..

[33] Deshpande L.M., Fritsche T.R., Moet G.J., Biedenbach D.J., Jones R.N. (2007). Antimicrobial resistance and molecular epidemiology of vancomycin-resistant enterococci from North America and Europe: A report from the SENTRY antimicrobial surveillance program. Diagn. Microbiol. Infect. Dis..

[34] Sharifi Y., Hasani A., Ghotaslou R., Naghili B., Aghazadeh M., Milani M., Bazmany A. (2013). Virulence and Antimicrobial Resistance in Enterococci Isolated from Urinary Tract Infections. Adv. Pharm. Bull..

[35] Fontana R., Aldegheri M., Ligozzi M., Lopez H., Sucari A., Satta G. (1994). Overproduction of a low-affinity penicillin-binding protein and high-level ampicillin resistance in *Enterococcus faecium*. Antimicrob. Agents Chemother..

[36] Zapun A., Contreras-Martel C., Vernet T. (2008). Penicillin-binding proteins and β-lactam resistance. FEMS Microbiol. Rev..

[37] Arias C., Contreras G., Murray B. (2010). Management of multidrug-resistant enterococcal infections. Clin. Microbiol. Infect..

[38] Wu X., Hou S., Zhang Q., Ma Y., Zhang Y., Kan W., Zhao X. (2016). Prevalence of virulence and resistance to antibiotics in pathogenic enterococci isolated from mastitic cows. J. Vet.-Med. Sci..

[39] Miller W.R., Munita J.M., Arias A.C. (2014). Mechanisms of antibiotic resistance in enterococci. Expert Rev. Anti-Infect. Ther..

[40] WHO (2015). Global Action Plan on Antimicrobial Resistance 2017.

[41] Cortés C., Delafuente R., Contreras A., Sánchez A., Corrales J., Ruizsantaquiteria J., Orden J. (2006). Occurrence and preliminary study of antimicrobial resistance of enterococci isolated from dairy goats in Spain. Int. J. Food Microbiol..

[42] Mannu L., Paba A., Daga E., Comunian R., Zanetti S., Duprè I., Sechi L. (2003). Comparison of the incidence of virulence determinants and antibiotic resistance between *Enterococcus faecium* strains of dairy, animal and clinical origin. Int. J. Food Microbiol..

[43] Ahmed M.O., Baptiste K.E. (2018). Vancomycin-Resistant Enterococci: A Review of Antimicrobial Resistance Mechanisms and Perspectives of Human and Animal Health. Microb. Drug Resist..

[44] Xanthopoulou K., Peter S., Tobys D., Behnke M., Dinkelacker A.G., Eisenbeis S., Falgenhauer J., Falgenhauer L., Fritzenwanker M., Gölz H. (2020). Vancomycin-resistant *Enterococcus faecium* colonizing patients on hospital admission in Germany: Prevalence and molecular epidemiology. J. Antimicrob. Chemother..

[45] ECDC (2019). Surveillance of Antimicrobial Resistance in Europe 2018.

[46] Martín-Platero A.M., Valdivia E., Maqueda M., Martínez-Bueno M. (2009). Characterization and safety evaluation of enterococci isolated from Spanish goats’ milk cheeses. Int. J. Food Microbiol..

[47] Song H., Bae Y., Jeon E., Kwon Y., Joh S. (2019). Multiplex PCR analysis of virulence genes and their influence on antibiotic resistance in *Enterococcus* spp. isolated from broiler chicken. J. Vet.-Sci..

[48] Perin L.M., Miranda R.O., Todorov S.D., Franco B.D.G.d.M., Nero L.A. (2014). Virulence, antibiotic resistance and biogenic amines of bacteriocinogenic lactococci and enterococci isolated from goat milk. Int. J. Food Microbiol..

[49] Jackson C.R., Fedorka-Cray P.J., Barrett J.B. (2004). Use of a Genus- and Species-Specific Multiplex PCR for Identification of Enterococci. J. Clin. Microbiol..

[50] Barcelos M.M., Martins L., Grenfell R.C., Juliano L., Anderson K.L., dos Santos M.V., Gonçalves J.L. (2019). Comparison of standard and on-plate extraction protocols for identification of mastitis-causing bacteria by MALDI-TOF MS. Braz. J. Microbiol..

[51] Peltroche-Llacsahuanga H., Top J., Weber-Heynemann J., Lütticken R., Haase G. (2009). Comparison of Two Chromogenic Media for Selective Isolation of Vancomycin-Resistant *Enterococci* from Stool Specimens. J. Clin. Microbiol..

[52] Bauer A.W., Kirby W.M.M., Sherris J.C., Turck M. (1966). Antibiotic Susceptibility Testing by a Standardized Single Disk Method. Am. J. Clin. Pathol..

[53] Vankerckhoven V., Van Autgaerden T., Vael C., Lammens C., Chapelle S., Rossi R., Jabes D., Goossens H. (2004). Development of a Multiplex PCR for the Detection of *asa1*, *gelE*, *cylA*, *esp*, and *hyl* Genes in Enterococci and Survey for Virulence Determinants among European Hospital Isolates of *Enterococcus faecium*. J. Clin. Microbiol..

[54] Eaton T.J., Gasson M.J. (2001). Molecular Screening of *Enterococcus* Virulence Determinants and Potential for Genetic Exchange between Food and Medical Isolates. Appl. Environ. Microbiol..

[55] Creti R., Imperi M., Bertuccini L., Fabretti F., Orefici G., Di Rosa R., Baldassarri L. (2004). Survey for virulence determinants among *Enterococcus faecalis* isolated from different sources. J. Med. Microbiol..

